# Is perioperative colloid infusion more effective than crystalloid in preventing postoperative nausea and vomiting? A systematic review and meta-analysis

**DOI:** 10.1097/MD.0000000000014339

**Published:** 2019-02-15

**Authors:** Hyun Jung Kim, Seung Ho Choi, Darhae Eum, Seung Hyun Kim

**Affiliations:** a Institute for Evidence-based Medicine, Department of Preventive Medicine, College of Medicine, Korea University; b Department of Anesthesiology and Pain Medicine, Anesthesia and Pain Research Institute, Severance Hospital; c Department of Anesthesiology and Pain Medicine, Severance Hospital, Yonsei University College of Medicine, Seoul, Korea.

**Keywords:** colloid, colloid, crystalloid, crystalloid, general anesthesia, general anesthesia, meta-analysis, postoperative nausea and vomiting, postoperative nausea and vomiting

## Abstract

**Background:**

Adequate intravenous fluid replacement is recommended as an effective nonpharmacologic strategy for reducing postoperative nausea and vomiting (PONV), one of the most common and stressful complications of general anesthesia. We aimed to evaluate the effect of hydration, according to the type of fluid, on PONV as previous studies have reported inconsistent results.

**Methods:**

We performed a systemic review and meta-analysis of randomized controlled trials (RCTs) comparing infusion of colloid with that of crystalloid in terms of PONV incidence and the need for rescue antiemetic therapies for 24 hours after surgery under general anesthesia. The effect of fluid infusion according to the duration of anesthesia was also examined. A literature search was performed, using MEDLINE, Excerpta Medica Database, Cochrane Central Register of Controlled Trials, Web of Science, and Scopus, up to February 2018.

**Results:**

We included 8 RCTs. Compared with the crystalloid infusion, perioperative colloid infusion did not reduce PONV incidence, with a relative risk of 0.87 (95% confidence interval [CI], 0.60–1.25). However, subgroup analysis by duration of anesthesia showed a statistically significant subgroup effect (*P = *.04, *I*^*2*^ = 77.4%), suggesting that the effect of colloid differed from that of crystalloid depending on the duration of anesthesia. In the subgroup that underwent anesthesia for more than 3 hours, in which the patients had mostly undergone abdominal surgeries, colloid infusion significantly reduced the incidence of PONV compared with crystalloid infusion (RR, 0.69; 95% CI, 0.53–0.89). In the subgroup that underwent anesthesia for <3 hours, colloid infusion did not reduce the incidence of PONV compared with crystalloid infusion (RR, 1.32; 95% CI, 0.76–2.27). The requirement for antiemetics was comparable between colloid and crystalloid infusions, with an RR of 0.93 (95% CI, 0.55–1.58).

**Conclusion:**

Colloid administration had a more preventive effect on PONV than crystalloid administration in patients undergoing abdominal surgery under general anesthesia for more than 3 hours but did not show a preventive effect in patients undergoing anesthesia for <3 hours.

## Abstract 2

Supplemental Digital Content is available in the text

## 1 Introduction

Postoperative nausea and vomiting (PONV) is one of the most common and stressful complications related to general anesthesia,^[1,2]^ and can cause patients’ dissatisfaction with their anesthesia care.^[3–5]^ Currently, many antiemetic medications are used to prevent PONV. However, universal pharmacological PONV prophylaxis is accompanied by side effects, such as oversedation, hypotension, dry mouth, and dysphoria, and is less cost-effective.^[6,7]^

A number of factors, including female sex, nonsmoking, general anesthesia, and duration of anesthesia, have been identified as independent risk factors for PONV.^[8]^ Especially most patients undergoing surgery are prone to gut ischemia because of overnight fasting prior to general anesthesia, and this could be associated with PONV.^[6]^ High volume of perioperative fluid administration has been shown to reduce the incidence of PONV in previous studies.^[9–12]^ Adequate intravenous fluid hydration has been recommended as an effective strategy for reducing the baseline risk for PONV in a previous consensus guideline.^[8]^ However, the effect of hydration, according to the type of fluid, on PONV is controversial.

Therefore, we conducted a meta-analysis with the aim of comparing the effectiveness of colloid infusion with that of crystalloid infusion in preventing PONV, using multiple randomized controlled trials (RCTs).

## 2 Materials and methods

We used multiple comprehensive databases to search for literature comparing the effect of colloid infusion with that of crystalloid infusion on the incidence of PONV. This study is based on the Cochrane Review Methods, and followed the “preferred reporting items for systematic reviews and meta-analyses” guidelines for reporting analyses. Ethical approval was not necessary because this is a review of previously published articles.

### 2.1 Data and literature source

We searched MEDLINE, Excerpta Medica Database, the Cochrane Central Register of Controlled Trials, Web of Science, and Scopus in February 2018. We applied no restrictions on language or year of publication in our search.

The following keywords and Medical Subject Headings were searched through MEDLINE: postoperative nausea and vomiting, colloid, starch, and anesthesia. See Supplementary Information 2 for the comprehensive list. Search strategies based on the MEDLINE strategy were adapted for the other databases. After the initial electronic search, we manually searched for further relevant articles among the identified studies. Identified articles were assessed for inclusion individually.

### 2.2 Study selection

The inclusion of all studies was independently decided by 2 authors (SHK and HJK) based on the selection criteria. Study selection was performed through 2 levels of screening. At the first level, we screened titles and abstracts of the identified studies. At the second level, we screened the full text. Studies were included in our meta-analysis if they met the following inclusion criteria: study population, adult patients undergoing surgery under general anesthesia; intervention, colloid fluid; control, crystalloid fluid; outcome measure, the incidence of PONV for 24 hours after the surgery, and the rescue antiemetic requirement for 24 hours after the surgery; and study design, prospective trials and RCTs. Duplicate publications, review articles, and case reports were excluded.

### 2.3 Data extraction

The 2 authors (SHK and HJK) independently extracted data from each study using a predefined data extraction form. Any disagreement unresolved by discussion was reviewed by a third author (SHC).

The following variables were extracted from the studies: first author; publication year; baseline characteristics of patients; type of surgery; intervention of fluid replacement; and outcome results, including the incidence of PONV, incidence of vomiting, and number of patients who needed rescue antiemetics for 24 hours after the surgery.

### 2.4 Assessment of methodological quality

Two authors (SHK and HJK) independently assessed the methodological qualities for each study using the Cochrane Collaboration's tool for assessing risk of bias. We assessed the following domains: random sequence generation, allocation concealment, blinding of participants and personnel, blinding of outcome assessment, incomplete outcome data, and selective reporting.

Publication bias was not assessable for these trials. Tests for funnel plot asymmetry are generally only performed when at least 10 studies are included in the meta-analysis. Since our analysis only includes 8 studies, tests for asymmetry would be ineffective as they would be unable to differentiate chance from asymmetry.

### 2.5 Statistical analysis

The primary outcome of our meta-analysis was the incidence of PONV for 24 hours after the surgery, and secondary outcomes were the incidence of postoperative vomiting and antiemetic requirements for 24 hours after the surgery. For dichotomous outcomes (incidence of PONV, postoperative vomiting, and antiemetic requirements), the results were expressed as relative risks (RR) with 95% confidence intervals (CI). Data were pooled using a random-effects model.

To estimate heterogeneity, we calculated the proportion of between-study inconsistency due to true differences between studies (rather than differences due to random error or chance) using the *I*^*2*^ statistic, with values of 25%, 50%, and 75% considered low, moderate, and high, respectively. We conducted subgroup analyses for situations where this might affect the results (duration of anesthesia). All analyses were performed using RevMan version 5.2 (The Cochrane Collaboration, London, UK).

Subgroup analyses were performed according to the duration of anesthesia. Three and 5 studies included patients undergoing anesthesia for more and less than 3 hours, respectively. Therefore, we divided the trials into 2 subgroups with long and short durations of anesthesia. Subgroup analyses were also performed according to other well-known risk factors for PONV, including female sex, nitrous oxide use, postoperative opioid, and type of surgery.

## 3 Results

### 3.1 Identification of studies

**Figure 1:**
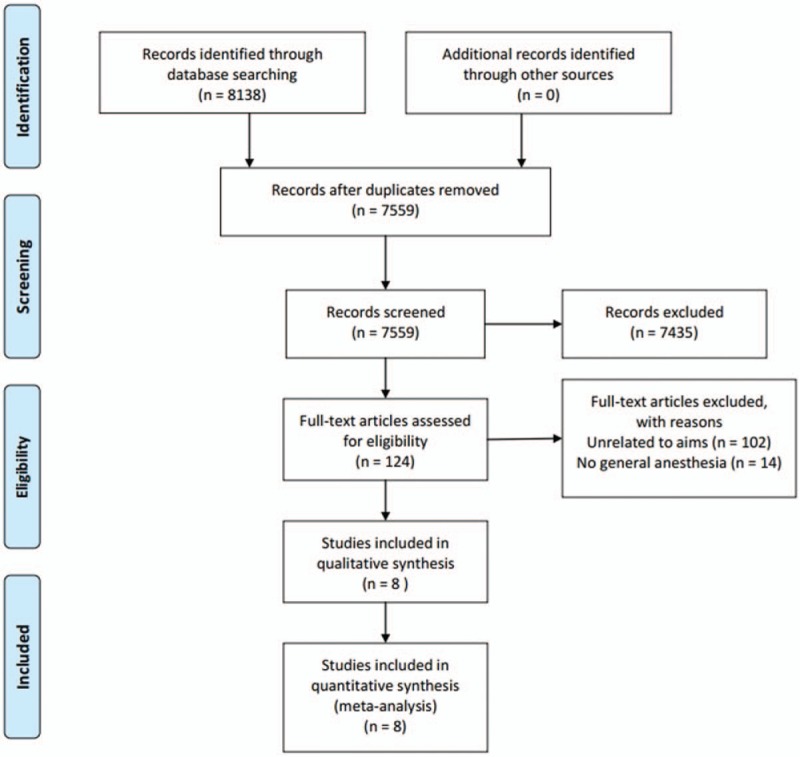
Meta-analysis flow-chart. RCT = randomized controlled trials.

Searches of all the databases resulted in 8138 articles. Of these, 579 publications were excluded as they were duplicated, 7435 publications were excluded as it was clear from the title and abstract that they did not fulfill the selection criteria. For the remaining 124 articles, we obtained full manuscripts, and following scrutiny of these, we identified 8 potentially relevant studies; 14 articles were excluded as they did not perform general anesthesia. Therefore, the total number of studies included in the review was 8 (Fig. 1).^[6,13–19]^

### 3.2 Study characteristics and patient populations

The 8 studies enrolled a total of 756 patients who underwent surgery under general anesthesia (Table 1). Overall, 343 patients were assigned randomly to the crystalloid group, and the remaining 413 patients were assigned to the colloid group. Four studies included only female patients, and another 4 studies included both male and female patients.

**Table 1:**
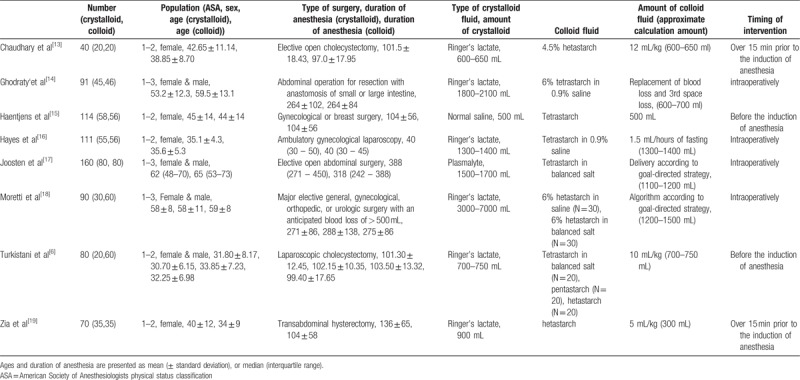
The main characteristics of the studies included in the meta-analysis.

All patients in both the groups were administered crystalloid as a maintenance fluid, and the interventional fluids were administered according to the study regimen. All patients in the colloid group received hydroxyethyl starch as an interventional fluid (3 studies used hetastarch; 4 studies used tetrastarch; and 1 study used hetastarch, pentastarch, and tetrastarch). The interventional fluid for the crystalloid group was Ringer's lactate in 6 studies, normal saline in 1 study, and plasmalyte in 1 study.

In the studies by Joosten et al^[17]^ and Moretti et al,^[18]^ the volume of interventional fluid was determined using a goal-directed fluid therapy. In a study by Ghodraty et al,^[14]^ intraoperative blood loss was replaced either with colloid at 1:1 ratio or with crystalloid at 3:1 ratio. The administered volume of interventional fluid in these 3 studies with patients undergoing anesthesia for more than 3 hours was significantly higher in the crystalloid group than in the colloid group. In a study by Zia et al,^[19]^ the volume of interventional fluid was 15 mL/kg for the crystalloid group, and 5 mL/kg for the colloid group. In the other 4 studies, the volume of crystalloid and colloid fluids were the same.

The timing of intervention varied according to the studies. In all 3 studies with anesthesia duration longer than 3 hours, interventional fluids were administered intraoperatively. On the other hand, among studies with anesthesia duration <3 hours, intervention was performed before induction of anesthesia in 4 studies, and intraoperatively in only one study (by Hayes et al^[16]^).

Four studies^[13,15–16,19]^ only included female patients, whereas another 4 studies^[6,14,17–18]^ included both male and female patients. One study by Ghodraty et al^[14]^ was performed under total intravenous anesthesia using propofol, whereas all other studies used inhalation anesthetics. Nitrous oxide and air were used in 3^[13,18–19]^ and 4 studies,^[6,14–16]^ respectively. Patients in 2 studies^[16,19]^ received postoperative opioids when needed, but patients in 4 studies^[6,13–15]^ did not receive it. The studies by Chaudhary et al^[13]^ and Turkistani et al^[6]^ only included patients without history of smoking, motion sickness, and previous PONV, and 4 studies^[6,13,15,19]^ only included patients who did not receive prophylactic antiemetics.

Two studies were performed in Belgium, and the remaining 6 studies were performed in the United States, Ireland, Saudi Arabia, Iran, Pakistan, and India, respectively.

### 3.3 Quality of the included studies

**Figure 2:**
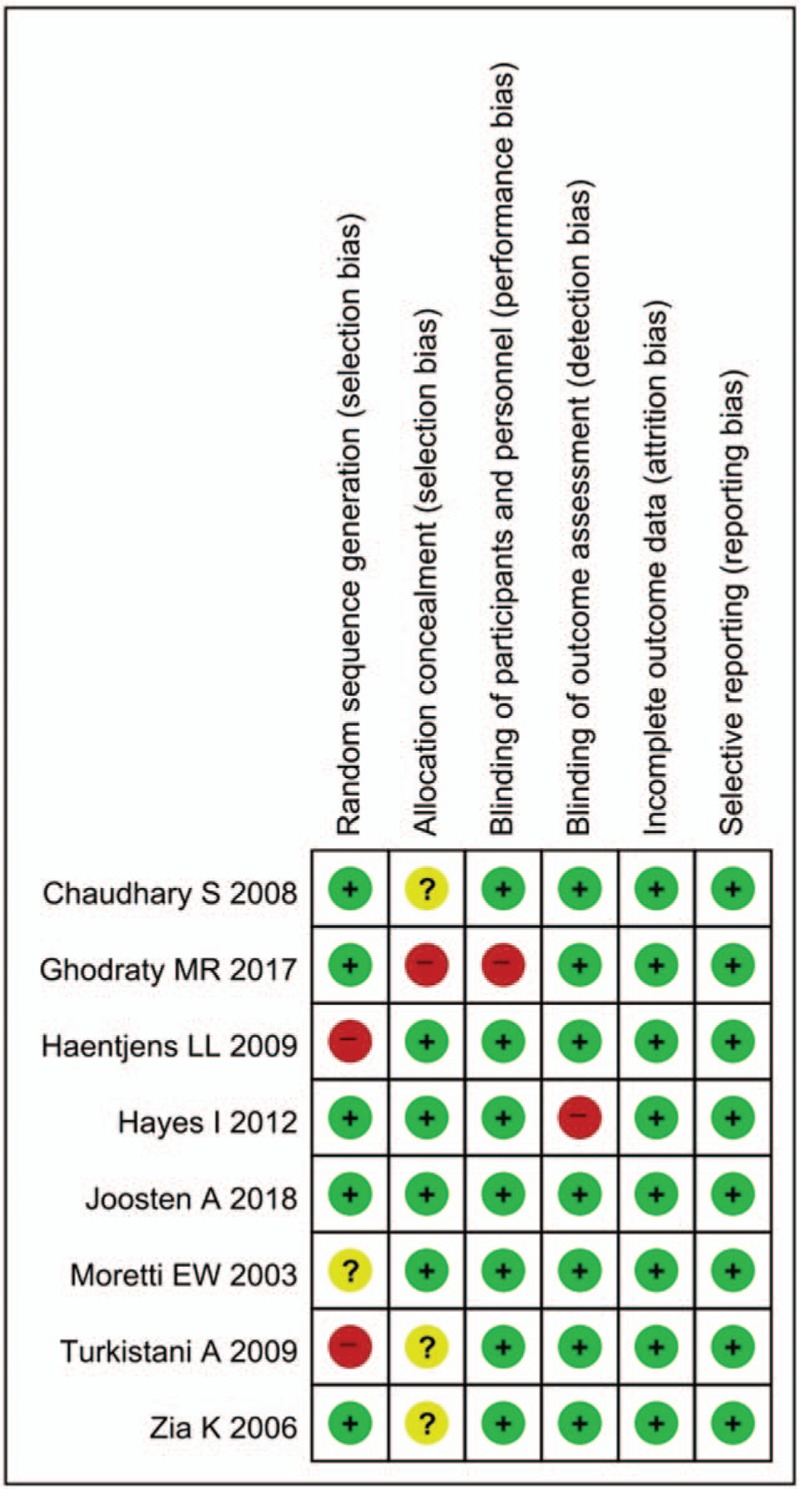
Risk of bias of original studies.

We have reported the risk of bias in Figure 2. One study by Ghodraty et al^[14]^ was at high risk with respect to the allocation and blinding of participants. However, given the difficulty of additional intervention, these risks are unlikely to have affected the further intervention during anesthesia and the outcome assessment.

### 3.4 Incidence of PONV and antiemetics requirement

**Figure 3:**
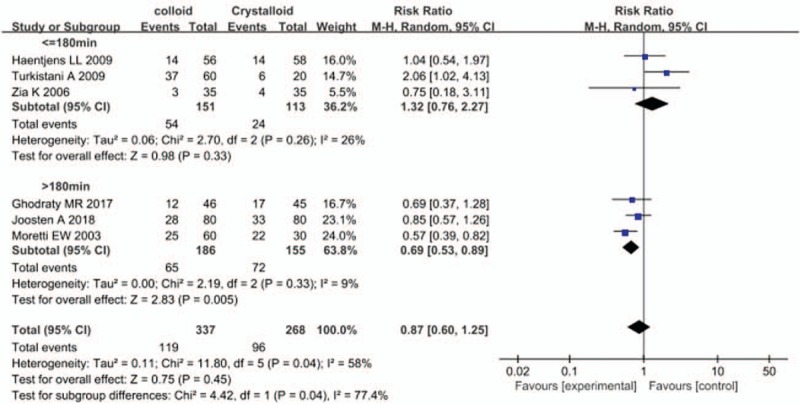
Forest plot of the effects of perioperative intravenous colloid infusion on the incidence of postoperative nausea and vomiting, according to the duration of anesthesia. CI = confidence interval.

**Figure 4:**
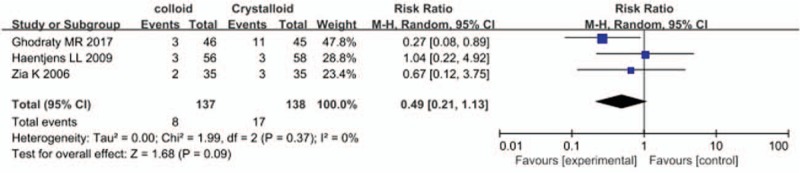
Forest plot of the effects of perioperative intravenous colloid infusion on the incidence of postoperative vomiting. CI = confidence interval.

**Figure 5:**
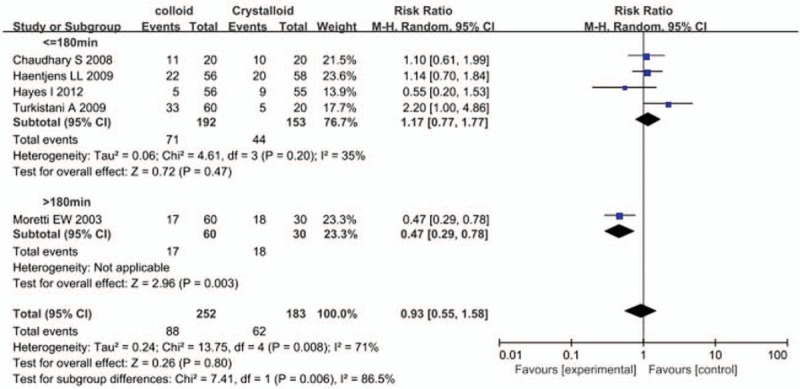
Forest plot of the effects of perioperative intravenous colloid infusion on the requirement for antiemetic therapy, according to the duration of anesthesia. CI = confidence interval.

Six studies reported the incidence of PONV, and perioperative colloid infusion showed a trend of reducing PONV compared with crystalloid infusion; however, the difference was not statistically significant (RR, 0.87; 95% CI, 0.60–1.25) (Fig. 3). The results of 3 studies also showed that colloid infusion did not reduce the incidence of vomiting (RR, 0.49; 95% CI, 0.21–1.13) (Fig. 4). Similarly, 5 studies found no difference in the antiemetics requirement between the colloid and crystalloid groups (RR, 0.93; 95% CI, 0.55–1.58) (Fig. 5).

### 3.5 Subgroup analysis by duration of anesthesia

A subgroup analysis stratified by duration of anesthesia demonstrated that perioperative colloid infusion could effectively prevent PONV associated with anesthesia duration of more than 3 hours compared with crystalloid infusion. A total of 341 patients were included in 3 studies of major surgeries, and 279 patients (81.8%) among them underwent abdominal surgeries. The remaining 62 patients underwent gynecological, orthopedic, or urologic surgeries. The results of this subgroup showed that colloid infusion significantly reduced the incidence of PONV compared with crystalloid infusion (RR, 0.69; 95% CI, 0.53–0.89). Only one study by Morettei et al^[18]^ reported the requirement for antiemetics, which was also reduced in the colloid group compared with the crystalloid group (RR, 0.47; 95% CI, 0.29–0.78).

In contrast, perioperative colloid infusion did not improve the incidence of PONV in studies with anesthesia duration <3 hours. A total of 415 patients were included in 5 such studies. The patients in this subgroup underwent breast and gynecological surgeries and cholecystectomy, which included both transabdominal and laparoscopic approaches. In 3 studies, colloid infusion did not reduce the incidence of PONV compared with crystalloid infusion (RR, 1.32; 95% CI, 0.76–2.27). Similarly, 4 studies found no difference in the requirement for antiemetics between the colloid and crystalloid groups (RR, 1.17; 95% CI, 0.77–1.77).

### 3.6 Subgroup analysis of studies including only female patients

**Figure 6:**
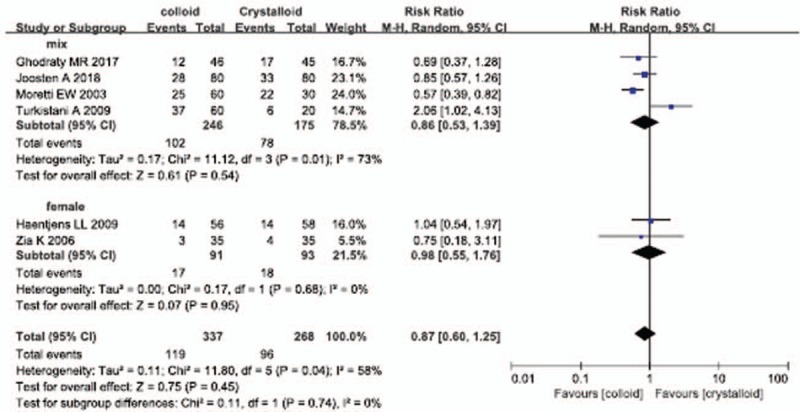
Forest plot of the effects of perioperative intravenous colloid infusion on the incidence of postoperative nausea and vomiting, according to sex. CI = confidence interval.

In the subgroup of female-only studies, colloid administration did not reduce the incidence of PONV compared with crystalloid infusion (RR, 0.98; 95% CI, 0.55–1.76) (Fig. 6). Moreover, there was no subgroup difference in the incidence of PONV between the female-only and mixed (including both male and female patients) studies (*P = *.74).

### 3.7 Subgroup analysis by nitrous oxide use

In studies with anesthesia duration <3 hours, there was no difference in the incidence of PONV between the subgroups with nitrous oxide and air use (*P = *.42) (Supplementary Fig. 1). Further, in studies with anesthesia duration more than 3 hours, there was no difference in the incidence of PONV between the 2 subgroups (*P = *.59) (Supplementary Fig. 2).

### 3.8 Subgroup analysis by postoperative opioid use

There was no difference in the incidence of PONV between the subgroups with and without opioid use (*P = *.42) (Supplementary Fig. 3).

### 3.9 Subgroup analysis by type of surgery

In the subgroup of open abdominal surgeries, colloid infusion did not reduce the incidence of PONV compared with crystalloid infusion (RR, 0.80; 95% CI, 0.57–1.11) (Supplementary Fig. 4). There was no difference in the incidence of PONV between the subgroups with open abdominal surgeries and open hysterectomy (*P = *.93).

## 4 Discussion

In this meta-analysis, we found that the effect of perioperative colloid administration showed a clear difference according to duration of anesthesia (subgroup difference, *I*^*2*^ = 77.4%). Therefore, the effect of colloid infusion on PONV should be interpreted based on the duration of anesthesia, and it seems to exert more preventive effect against PONV than crystalloid infusion in patients undergoing abdominal surgery under general anesthesia for more than 3 hours.

As a routine, patients are advised to fast overnight before elective surgery. Due to overnight fasting and intraoperative surgical losses that often cannot be replaced adequately, hypovolemia results in decreased blood flow to the gastrointestinal tract.^[13]^ When the circulating volume decreases, blood is redistributed from the splanchnic bed to more vital organs, such as the brain and kidney.^[20]^ Perioperative volume expansion via colloid infusion is known to improve the perfusion of the gut mucosa,^[21]^ and adequate intravenous hydration is recommended as an effective strategy for reducing the baseline risk for PONV.^[8,10]^ According to the previous studies, the administration of large amounts of crystalloids reduces the incidence of PONV in patients under general anesthesia compared with smaller amounts.^[12,15,22]^ Based on this meta-analysis, the effectiveness of crystalloid infusion was comparable with that of colloid infusion in preventing PONV following general anesthesia for <3 hours, and this may be related to the volume effect.

In 3 studies with anesthesia duration more than 3 hours, 279 (81.8%) out of 341 patients underwent abdominal surgeries. Certain types of surgeries, including abdominal surgeries, that require long duration of general anesthesia and high opioid consumption due to severe pain are associated with high incidence of PONV.^[8]^ In this review, the incidence of PONV in patients under anesthesia for more than 3 hours was significantly higher than that in patients under anesthesia for <3 hours (137/341 [40.2%] vs 78/264 [29.5%]; odds ratio, 1.60; 95% CI, 1.14–2.25; *P = *.01). In these high-risk patients under anesthesia for more than 3 hours, colloid infusion was more effective in reducing the incidence of PONV than crystalloid infusion.

It is difficult for colloids to cross the vascular endothelium because of their large molecular weight, and they remain in the intravascular space longer than crystalloids. According to a previous meta-analysis, lower fluid volumes are required to achieve similar hemodynamic end points using colloids than that using crystalloids,^[23]^ resulting in milder interstitial edema.^[18]^ Colloid administration could possibly reduce the postoperative ileus by reducing the gut edema.^[24]^ Particularly, most of the patients included in the subgroup of anesthesia duration more than 3 hours underwent abdominal surgeries, and the administered volume of interventional fluid was significantly higher in the crystalloid group than in the colloid group. In contrast, in the subgroup with anesthesia duration <3 hours, the administered volumes of interventional fluid were comparable between the 2 groups, except for a study by Zia et al^[19]^ These differences between the subgroups could be the reason for the variability in preventive effect of colloid infusion against PONV, and it is plausible that bowel-preserving effects of colloids may have reduced the incidence of PONV.

This review and meta-analysis has some limitations. Firstly, we included studies that varied with respect to the type of surgery and volume of administered interventional fluid, which may account for some of the heterogeneity observed in our analytical results. Secondly, since the studies included in this meta-analysis were mostly limited to cholecystectomy and gynecologic and abdominal surgeries, the results cannot be generalized to other surgeries. Further studies with larger numbers of patients and greater variety of surgeries are warranted to assess the beneficial effects of perioperative intravenous colloid infusion on PONV in patients under general anesthesia.

In conclusion, the effects of colloid infusion vary according to the duration of surgery, being more preventive against PONV than crystalloid infusion in patients undergoing abdominal surgery under general anesthesia for more than 3 hours. More studies are needed to determine the benefits of perioperative colloid infusion as a preventive measure against PONV.

## Author contributions

**Conceptualization:** Hyun Jung Kim, Seung Ho Choi, Seung Hyun Kim

**Data curation:** Hyun Jung Kim, Seung Ho Choi, Darhae Eum, Seung Hyun Kim

**Formal analysis:** Hyun Jung Kim, Seung Ho Choi, Darhae Eum, Seung Hyun Kim.

**Investigation:** Hyun Jung Kim, Seung Ho Choi, Darhae Eum, Seung Hyun Kim

**Methodology:** Hyun Jung Kim, Seung Ho Choi, Darhae Eum, Seung Hyun Kim

**Project administration:** Hyun Jung Kim, Seung Ho Choi, Darhae Eum, Seung Hyun Kim

**Resources:** Hyun Jung Kim, Seung Ho Choi, Darhae Eum, Seung Hyun Kim.

**Software:** Hyun Jung Kim, Seung Ho Choi, Darhae Eum, Seung Hyun Kim

**Supervision:** Hyun Jung Kim, Seung Ho Choi, Darhae Eum, Seung Hyun Kim.

**Validation:** Hyun Jung Kim, Seung Ho Choi, Darhae Eum, Seung Hyun Kim

**Visualization:** Hyun Jung Kim, Seung Ho Choi, Darhae Eum, Seung Hyun Kim

**Writing – original draft:** Hyun Jung Kim, Seung Hyun Kim

**Writing – review & editing:** Hyun Jung Kim, Seung Ho Choi, Darhae Eum, Seung Hyun Kim

Seung Hyun Kim orcid: 0000-0003-2127-6324.

## Supplementary Material

Supplemental Digital Content
