# Supplementary material for: Is perioperative colloid infusion more effective than crystalloid in preventing postoperative nausea and vomiting? A systematic review and meta-analysis
Source: Medicine (Baltimore). 2019 Feb 15;98(7):e14339. doi: 10.1097/MD.0000000000014339 (PMC6408100; doi:10.1097/MD.0000000000014339)

Supplementary Fig. 1. Forest plot of the effects of perioperative intravenous colloid infusion on the incidence of postoperative nausea and vomiting, according to nitrous oxide use in studies with anesthesia duration less than 3░hours. CI░=░confidence interval.


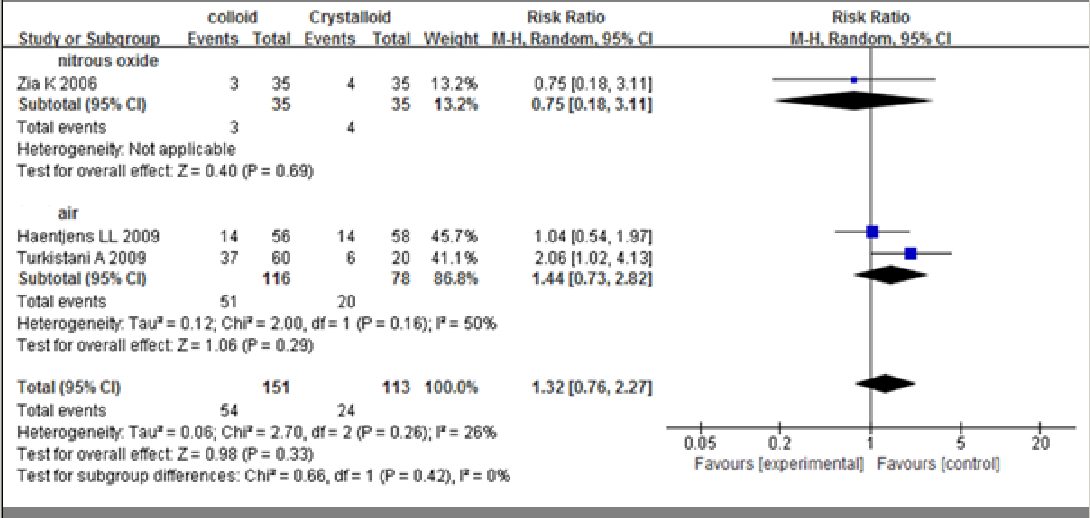


Supplementary Fig. 2. Forest plot of the effects of perioperative intravenous colloid infusion on the incidence of postoperative nausea and vomiting, according to nitrous oxide use in studies with anesthesia duration more than 3░hours. CI░=░confidence interval.


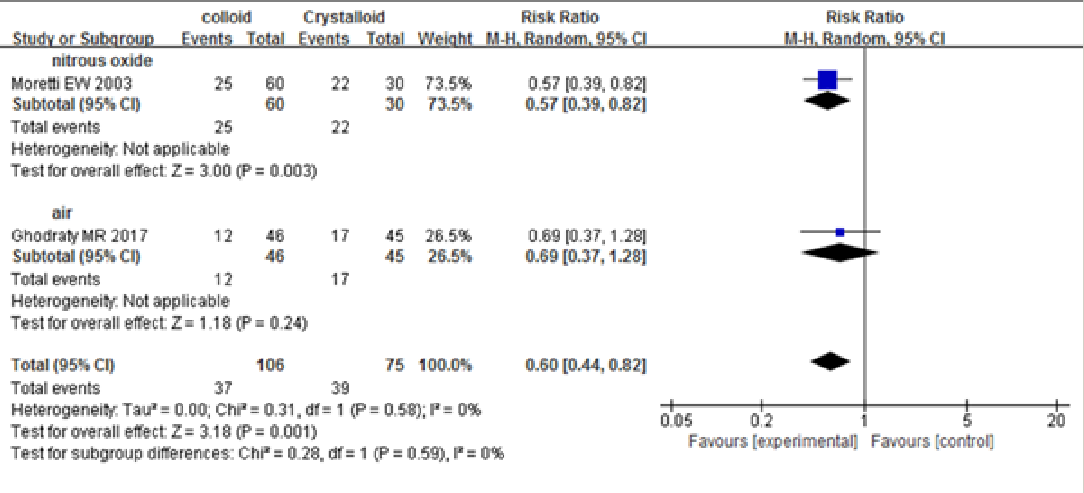


Supplementary Fig. 3. Forest plot of the effects of perioperative intravenous colloid infusion on the incidence of postoperative nausea and vomiting, according to postoperative opioid use. CI░=░confidence interval.


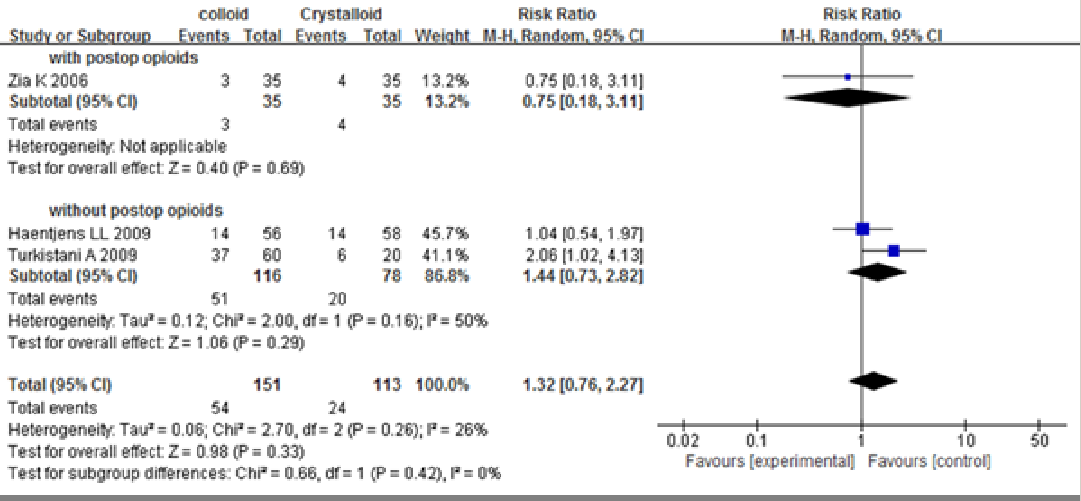


Supplementary Fig. 4. Forest plot of the effects of perioperative intravenous colloid infusion on the incidence of postoperative nausea and vomiting, according to type of surgery. CI░=░confidence interval.


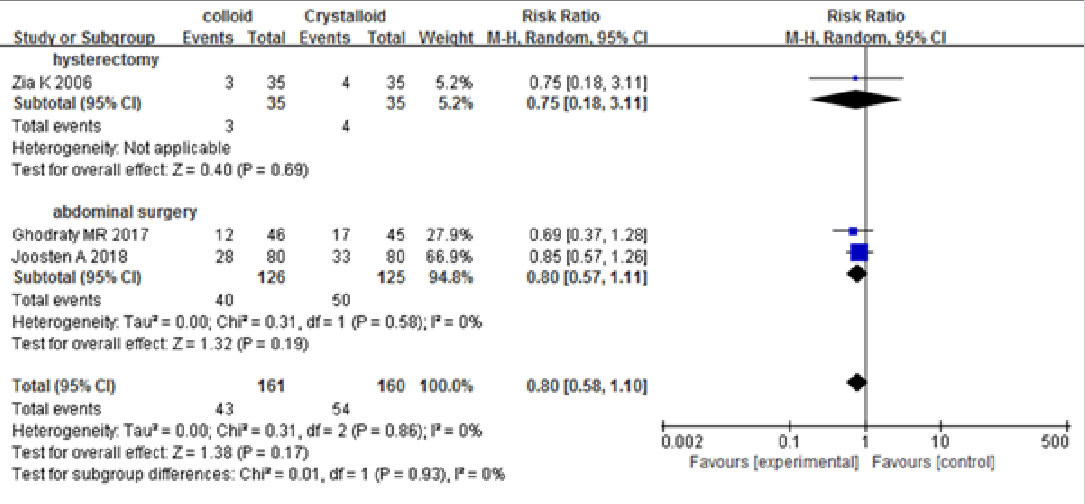

Supplement: Supplemental Digital Content [file medi-98-e14339-s001.doc]
